# Anomalous Blood Supply of the Superficial Face: A Case Report and Cadaveric Study of Facial and Transverse Facial Artery Variations

**DOI:** 10.7759/cureus.75526

**Published:** 2024-12-11

**Authors:** Brenton Stucki, Nicholas Jones, Dallin Judd, Braden Van Alfen, Rylan Fowers, Armando Rosales, Rehana S Lovely

**Affiliations:** 1 Texas College of Osteopathic Medicine, University of North Texas Health Science Center, Fort Worth, USA; 2 Joe R. and Teresa Lozano Long School of Medicine, University of Texas (UT) Health San Antonio, San Antonio, USA; 3 Center for Anatomical Sciences, University of North Texas Health Science Center, Fort Worth, USA; 4 Department of Physiology and Anatomy, University of North Texas Health Science Center, Fort Worth, USA

**Keywords:** anatomical case study, anatomy, face, facial vascular anomalies, head and neck surgery, maxillofacial surgery, otolaryngology, plastic surgery, surgery, surgical planning

## Abstract

The facial and transverse facial arteries supply blood to the superficial structures of the face. Understanding these arterial variations is essential for optimizing surgical planning and outcomes, especially in invasive facial procedures. A 78-year-old male cadaveric dissection documented variations in facial and transverse facial arteries. A hypoplastic facial artery arose from the external carotid artery between the lingual and occipital arteries and terminated below the oral fissure, supplying only the lower face. The transverse facial artery, originating from the superficial temporal artery, took an atypical path, running deep to the parotid gland and following the nasolabial sulcus to supply the upper face, areas typically served by the facial artery. The variations observed in the facial and transverse facial arteries highlight the diversity in facial vascular anatomy. Preoperative identification of such anomalies can help minimize surgical risks and improve outcomes, making detailed anatomical knowledge critical for tailoring surgical approaches.

## Introduction

The facial arteries (FAs) and transverse FAs (TFAs) provide the major arterial supply for the superficial face. The FA arises from the external carotid artery, in the carotid triangle of the neck, and crosses the angle of the mandible as it ascends the anterolateral aspect of the face [1]. Following a distinctive course, it continues by running along the oral commissure, giving off the superior and inferior labial branches. Continuing its trajectory, the artery ascends along the nasolabial sulcus, providing the lateral nasal branch, and concludes its course by terminating as the angular branch near the medial aspect of the eye [2]. The TFA, which arises from the superficial temporal artery, has a significant role in lateral face vascularization by supplying blood to the parotid gland, masseter, and integument and terminating near the buccal area [3].

Knowledge of the arterial supply of the superficial face is critical for plastic surgery procedures such as face flaps or lip reconstructions, as well as maxillofacial or trauma surgeries involving repair of the midface. An awareness of possible anatomical variations of this blood supply is critical in avoiding surgical complications, many of which result from TFA transection [4].

This case adds to the body of knowledge regarding variation in the origin and course of arteries in the superficial face. There have been a few documented cases similar to this one involving a hypoplastic FA and a more dominant TFA, one of which was found bilaterally [5], and the other of which was found as part of a larger study in only one of 192 subjects [3]. Knowledge of this unique but significant anatomical variation is notable and worthy of clinical consideration during surgical procedures involving the region.

This article was previously presented as a meeting abstract at the 2024 UNTHSC Research Appreciation Day on March 21, 2024.

## Case presentation

**Figure 1:**
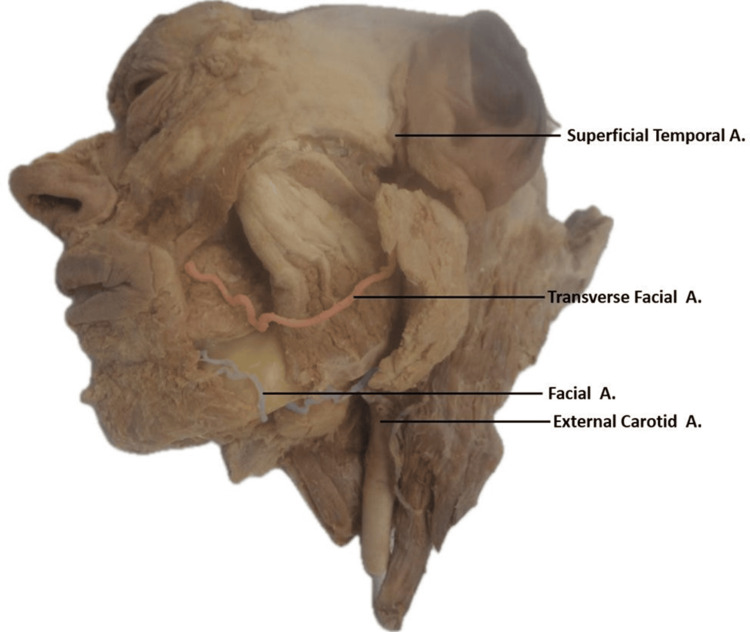
Course of the transverse facial artery and facial artery: path through parotid gland and nasolabial sulcus in left hemisected face. The course of the transverse facial artery (red) and facial artery (blue) under the parotid gland and following the nasolabial sulcus.

During a unilateral dissection of a 78-year-old donor, variations of the FAs and TFAs were observed. The superficial and deep structures of the face were dissected and cleaned to expose relevant structures, including the origins and terminations of the arteries of the face. The variant anatomical structures were noted and photographed. Specifically, it was noted that the TFA passed under the parotid gland across the lateral face before diving under the zygomaticus major and climbing the nasolabial sulcus, and the FA terminated prematurely (Figure 1).

**Figure 2:**
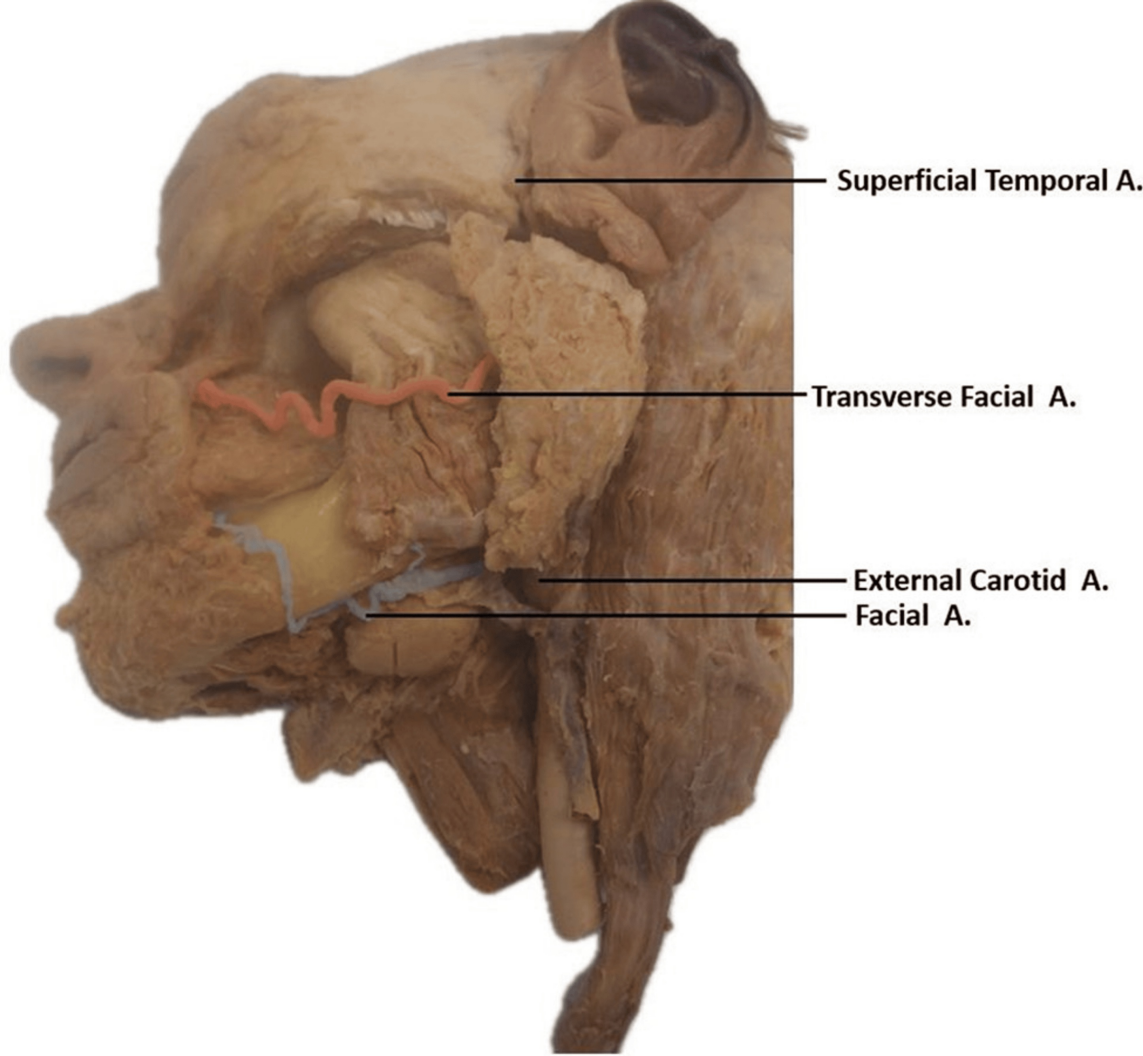
Path and termination of hypoplastic facial artery and hyperplastic transverse facial artery in left hemisected face. Left hemisected face illustrating the path and termination of the hypoplastic tortuous facial artery (blue) and the transverse path of the hyperplastic transverse facial artery (red).

The FA was found to arise from the external carotid artery, located between the lingual and occipital arteries. Unlike the usual course of the FA, this variant hypoplastic artery ended below the oral fissure after crossing the angle of the mandible (Figure 2). Its primary role appeared to be restricted to supplying blood to the lower structures of the face, as its tortuous pathway terminated prematurely before crossing above the level of the oral fissure (Figure 2).

**Figure 3:**
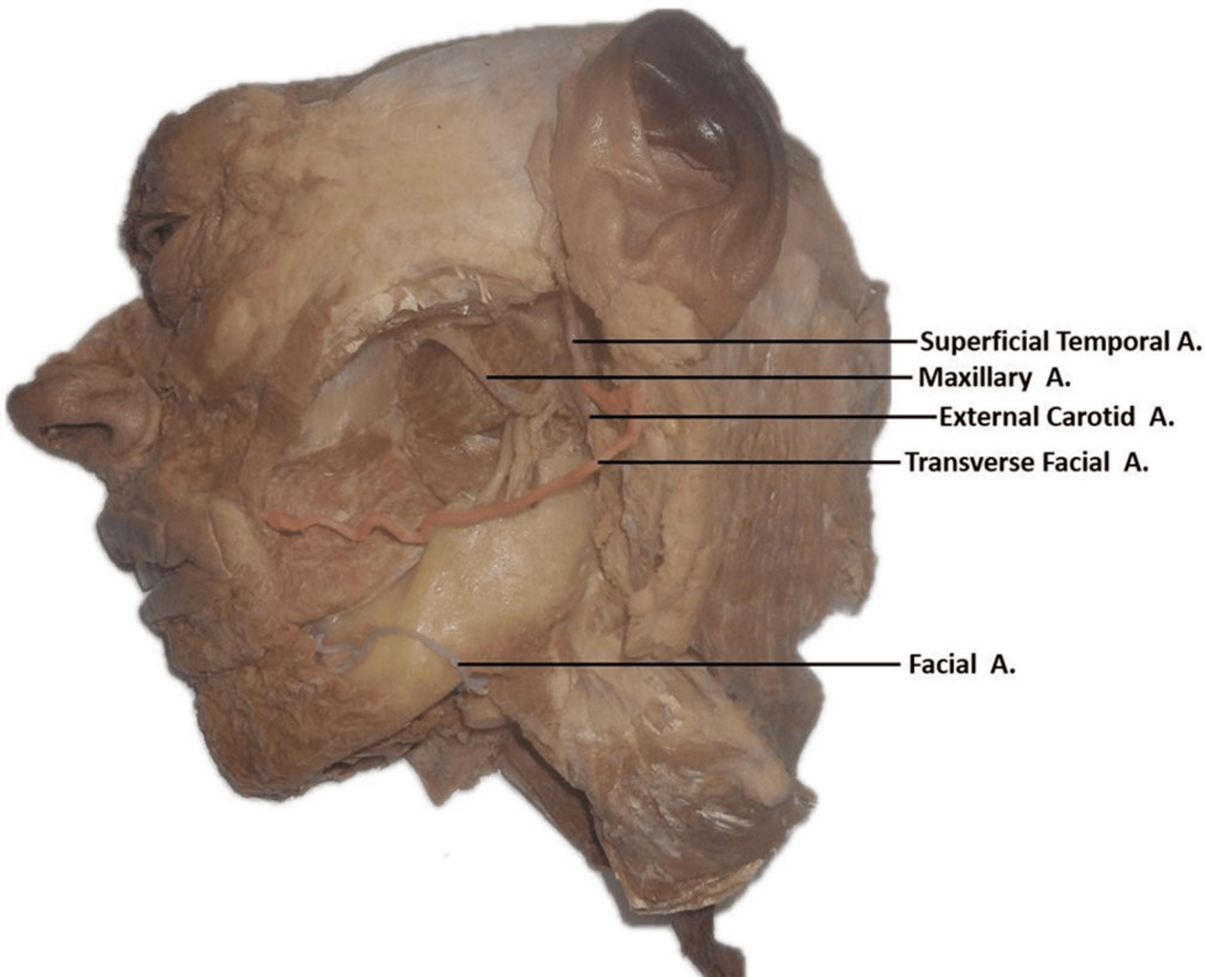
Branches of the external carotid artery with reflected parotid gland: origin of the facial and transverse facial arteries. Left hemisected face highlighting branches of the external carotid artery with the parotid gland reflected. The facial artery (blue) arises from the external carotid artery. The transverse facial artery (red) arises as a terminal branch of the external carotid artery.

In contrast, the TFA arises as a terminal branch of the external carotid artery. The TFA then runs deep to the parotid gland and extends across the lateral face (Figure 1). After reaching the nasolabial sulcus, it continued in the typical path that the FA usually takes. Following the nasolabial sulcus superiorly, the TFA is seen supplying the muscles and tissues above the oral fissure. It was observed that this variant transverse artery supplied blood to the majority of the superficial face instead of the FA (Figure 3).

## Discussion

The TFA and the FA are derivatives of the external carotid artery [6]. During the fourth and fifth weeks of embryological development, the aortic sac yields the aortic arches. It is then that the external carotid develops from the third aortic arch and directly gives rise to the FA [7]. During development, the external carotid artery gives rise to the superficial temporal artery, which provides blood supply to the lateral face as well through its branch, the TFA [3]. The timing and sequences of growth and development of the FAs influence the final configuration and branching patterns of the arterial network supplying the superficial face, resulting in either typical anatomy or anatomical variations.

The FA arises in the carotid triangle, arising beneath the platysma before continuing superficially [4]. The FA typically follows its tortuous path over the lingual artery descending the ramus of the mandible and continues superiorly as it runs obliquely across the cheek nearing the oral commissure and ascends the nasal fold to the medial canthus of the eye [8].

The termination of the FA can vary; a large meta-analysis of both cadaveric and computed tomography angiography (CTA) studies showed the prevalence of FA terminations as follows: superior or inferior labial (15.55%), inferior alar (5.89%), lateral nasal or angular (69.81%), dominant lateral branch (1.72%), and hypoplastic FA (0.81%) [9]. This indicates that although many variations are significantly prevalent, in most cases, the FA terminates in the area of the lateral nose and upper lip.

In a related study conducted by Koziej et al., the prevalence of various configurations of the TFA obtained by CTA was outlined in 100 patients, yielding 200 total cases analyzed [3]. This study does not have the same predictive power as the aforementioned meta-analysis on the FA, but it is the largest available data set on TFA configurations. In the vast majority of cases reported in this study, the TFA originated from the superficial temporal artery (91.7%). The other configurations found were that the TFA originated from the external carotid before its bifurcation into the superficial temporal and maxillary arteries (3.1%), or that the TFA originated from a trifurcation of the external carotid, the other two terminations being the superficial temporal and maxillary arteries. It was in one of these cases that a dominant TFA was found over a hypoplastic FA, as in our case [3].

The case presented in this report is not typical based on the current available literature. A hypoplastic FA dominated by a hyperplastic TFA has only been recorded a small number of times [3,5]. This rare configuration is of great clinical significance and should be accounted for in surgeries such as plastic, maxillofacial, or reconstructive surgeries involving the region. Some surgeries including lip reconstructions or facelift flaps utilize and surgically manipulate the terminations of the FA and TFA. In a case such as ours, significant surgical planning would be beneficial to avoid complications, as the course of action deviates greatly compared to a case involving the most common anatomical configurations. This outlines a possible benefit of preoperative imaging such as CTA in the planning of oral maxillofacial surgeries or procedures on the superficial face. While preoperative imaging such as this may offer potential benefits for identifying variant anatomy, its routine use in clinical settings is often constrained by cost, limited availability, and the risk-to-benefit ratio for patients [10].

In the context of the observed anatomical variations discussed, the deviation and distribution of the blood supply of the superficial face underscore the plasticity and adaptability of a developing vascular system [11]. Additionally, a study regarding the divergences of FA terminations has revealed potential arterial compensations for the FA’s shortness, indicating the clinical relevance of such variations discussed [12].

Understanding possible anatomical variations is crucial in clinical and surgical practices, particularly in plastic and reconstructive surgery of the face and neck, in cases of trauma or congenital malformations [13]. Embryonic signaling pathways, genetic factors, and environmental influences contribute to the variability seen in arterial development [14]. Embryological theories such as the axial sciatic theory (AST) as proposed by Al-Talalwah and Soames develop the argument that vascular variations result from development-related factors during embryogenesis [15]. These principles, while traditionally applied to axial arteries, may extend to craniofacial arteries, further heightening the importance of understanding vascular variability. Elucidation of such anomalies is critical for the reduction of iatrogenic complications and the enhancement of surgical planning. Specifically, knowledge of the possibility of variations is essential for reducing the risk of iatrogenic injuries such as damage to the superficial arteries of the face and flap necrosis in the event of specific procedures [16]. Preoperative imaging modalities coupled with an understanding of embryological principles could aid physicians in identifying variant arterial patterns and guide surgical decision-making [17].

## Conclusions

The face, like the rest of the human body, has a variety of documented anatomical variations. The observed anatomical variations of the FA and TFAs presented in this case highlight the complexity and diversity regarding the vascular supply of the face. Continued exploration of vascular anomalies enhances our understanding of facial vascular pathologies and can aid in facilitating diagnostic accuracy and inform surgical planning. Documentation of such anatomical variations enriches the collective knowledge base of facial vascular anatomy and informs medical education and clinical/surgical practice to help yield the best patient outcomes. Clarifying vascular variability will assist in the avoidance of iatrogenic faults. Therefore, radiologists, plastic surgeons, maxillofacial surgeons, vascular surgeons, and otolaryngologists must remain aware of the variability in vasculature and vascular anomalies.
